# The Mucosal Immune Function Is Not Compromised during a Period of High-Intensity Interval Training. Is It Time to Reconsider an Old Assumption?

**DOI:** 10.3389/fphys.2017.00485

**Published:** 2017-07-11

**Authors:** Dennis-Peter Born, Christoph Zinner, Billy Sperlich

**Affiliations:** Integrative and Experimental Exercise Science, Institute for Sport Sciences, University of Wuerzburg Wuerzburg, Germany

**Keywords:** circadian rhythm, cortisol, diurnal profile, endurance, high-volume training, immunoglobin-A, periodization

## Abstract

**Purpose:** The aim of the study was to evaluate the mucosal immune function and circadian variation of salivary cortisol, Immunoglobin-A (sIgA) secretion rate and mood during a period of high-intensity interval training (HIIT) compared to long-slow distance training (LSD).

**Methods:** Recreational male runners (*n* = 28) completed nine sessions of either HIIT or LSD within 3 weeks. The HIIT involved 4 × 4 min of running at 90–95% of maximum heart rate interspersed with 3 min of active recovery while the LSD comprised of continuous running at 70–75% of maximum heart rate for 60–80 min. The psycho-immunological stress-response was investigated with a full daily profile of salivary cortisol and immunoglobin-A (sIgA) secretion rate along with the mood state on a baseline day, the first and last day of training and at follow-up 4 days after the last day of training. Before and after the training period, each athlete's running performance and peak oxygen uptake (V^·^O_2peak_) was determined with an incremental exercise test.

**Results:** The HIIT resulted in a longer time-to-exhaustion (*P* = 0.02) and increased V^·^O_2peak_ compared to LSD (*P* = 0.01). The circadian variation of sIgA secretion rate showed highest values in the morning immediately after waking up followed by a decrease throughout the day in both groups (*P* < 0.05). With HIIT, the wake-up response of sIgA secretion rate was higher on the last day of training (*P* < 0.01) as well as the area under the curve (AUC_G_) higher on the first and last day of training and follow-up compared to the LSD (*P* = 0.01). Also the AUC_G_ for the sIgA secretion rate correlated with the increase in V^·^O_2peak_ and running performance. The AUC_G_ for cortisol remained unaffected on the first and last day of training but increased on the follow-up day with both, HIIT and LSD (*P* < 0.01).

**Conclusion:** The increased sIgA secretion rate with the HIIT indicates no compromised mucosal immune function compared to LSD and shows the functional adaptation of the mucosal immune system in response to the increased stress and training load of nine sessions of HIIT.

## Introduction

The exercise duration and intensity are key components to design training programs, stimulate adaptation and maximize performance of the modern day endurance athlete (Hydren and Cohen, 2015). Especially short intervals of 2–8 min at 90–95% of maximal heart rate (HR_max_) interspersed with periods of incomplete recovery known as high-intensity interval training (HIIT) are applied to maximize central and peripheral adaptation (Buchheit and Laursen, 2013a,b; Ronnestad et al., 2014, 2016; Stoggl and Sperlich, 2014, 2015; Sylta et al., 2016). When performing three training sessions per week for 8 weeks the HIIT (e.g., 4 × 4 min) significantly increased the peak oxygen uptake (V^·^O_2peak_) while no improvements occurred with the long-slow distance (LSD) running at 70% of HR_max_ for 45 min (Helgerud et al., 2007). The LSD sessions in the latter study however were fairly short while longer training sessions including low intensity have a small effect on V^·^O_2peak_ (Stoggl and Sperlich, 2014).

Still the superior benefits of HIIT to improve V^·^O_2peak_ have been shown multiple times in recreational and elite athletes among various endurance sports including running, cycling, rowing, and cross-country skiing (Helgerud et al., 2007; Ronnestad et al., 2014, 2016; Ni Cheilleachair et al., 2017). Despite the promising effects of HIIT however, the question arises among athletes, coaches and scientists whether the training load of several HIIT sessions within a short period of time might compromise the mucosal immune function. Especially the chronic exposure to high training loads has generally been assumed to increase the incidence of upper-respiratory tract infection (URTI; Trochimiak and Hubner-Wozniak, 2012).

A practical method to assess the stress-response from a psycho-immunological perspective during periods of intensified training is the combined assessment of the circadian variation of biomarkers in saliva and mood state (Papacosta et al., 2013; Born et al., 2016). While the concentrations of enzymes, hormones, and anti-bacterial compounds are far lower in saliva than in blood samples the relative changes in response to exercise are highly correlated to the blood serum (Cadore et al., 2008; VanBruggen et al., 2011; Tanner et al., 2014). The non-invasive collection of saliva allows a greater sampling rate and in an athletic population the entire circadian variation can be investigated with minimal interference in the daily training and recovery routines (Gatti and De Palo, 2011; Papacosta and Nassis, 2011).

Salivary immunoglobin-A (sIgA) and cortisol are biomarkers of particular interest when investigating the psycho-immunological stress-response during periods of intensified training. In ultra-marathon runners the extreme competition load acutely decreased the sIgA secretion rate and increased the levels of cortisol immediately after the exercise (Gill et al., 2014). Interestingly, low sIgA correlated with a high susceptibility of URTI and number of sick days during a period of polarized endurance training including both, continuous and interval training session (Ihalainen et al., 2016). Also an inverse correlation between levels of cortisol and sIgA secretion rate has been shown (Hucklebridge et al., 1998) and a potential decisive role of cortisol on the exercise-induced immune suppression during periods of intensified training discussed (Gleeson, 2007; He et al., 2010). Especially high-intensity exercise substantially increases the levels of cortisol (Allgrove et al., 2008). Therefore, the question arises whether the repeated exposure to HIIT over a prolonged period of time would compromise the mucosal immune function despite the tempting ergogenic effects including improved V^·^O_2peak_.

Earlier studies however mostly investigated the mucosal immune function with single pre- and post-training saliva samples (He et al., 2010; Gill et al., 2014; Ihalainen et al., 2016). Due to the dramatic decrease of cortisol and sIgA from early morning throughout the day (Hucklebridge et al., 1998; Rohleder et al., 2007), a full daily profile is warranted to investigate the circadian variation of these biomarkers of interest. Therefore, the aim of the study was to evaluate the mucosal immune function and circadian variation of salivary cortisol, sIgA secretion rate and mood during a period of nine sessions of HIIT performed within 3 weeks compared to LSD. The hypothesis was that the HIIT would increase levels of salivary cortisol, reduce sIgA secretion rate and impair mood thereby showing a more pronounced psycho-immunological stress-response and compromised mucosal immune function compared to the LSD.

## Methods

### Subject characteristics

For the present investigation, 28 recreational endurance runners were assigned into two groups performing either HIIT (*n* = 16, age: 25 ± 4 years, body mass: 76 ± 5 kg, body height: 179 ± 6 cm) or LSD (*n* = 12, age: 25 ± 3 years, body mass: 77 ± 11 kg, body height: 182 ± 5 cm). After being informed about the potential risks and benefits of the study involved, all runners gave their written consent to participate. The study was approved by the Ethical Committee of the University of Wuppertal and performed in accordance with the Declaration of Helsinki.

### Study design

Each athlete completed nine sessions of either HIIT or LSD within a period of 3 weeks with at least 1 day between the sessions (Stoggl and Sperlich, 2014) in addition to their routine aerobic training (4.0 ± 2.0 vs. 3.8 ± 1.6 h/week with the HIIT and LSD group, respectively) as performed previously (Ronnestad et al., 2014; Faiss et al., 2015). Before (Pre-) and after (Post-) the training period all participants performed an incremental test to exhaustion for the determination of running performance, i.e., time-to-exhaustion (TTE), as well as variables related to the cardio-respiratory and metabolic capacity. For each individual Pre- and Post- was scheduled on the same time of the day and performed with the same pair of running shoes within the same ambient air condition (20 ± 1°C and 36 ± 4% relative humidity).

Each HIIT session was initiated with a 10-min warm-up of moderate intensity running at 70% of maximum heart rate (HR_max_) including short bouts (30–45 s) with a higher running intensity to prepare the cardio-respiratory and metabolic system for the upcoming intervals. After the warm-up, the participants performed 4 × 4-min intervals with an exercise intensity corresponding to the individuals 90–95% of HR_max_ interspersed with 3 min of active recovery corresponding to 70% of HR_max_ (Helgerud et al., 2007). The HR data were recorded and each runner reached in at least 94% of all intervals (i.e., 34 of 36 possible intervals during the training period) the targeted exercise intensity of >90% of HR_max_ to be included in the statistical analysis. Due to the delayed HR response at the onset of exercise the athletes were instructed to reach the targeted HR zone (90–95% of HR_max_) within the first 60–90 s of each interval as recommended previously (Helgerud et al., 2007). The LSD was performed continuously at 70–75% of HR_max_. The duration for each LSD session was 60, 70, and 80 min for the first, second and third week of training, respectively.

**Figure 1:**
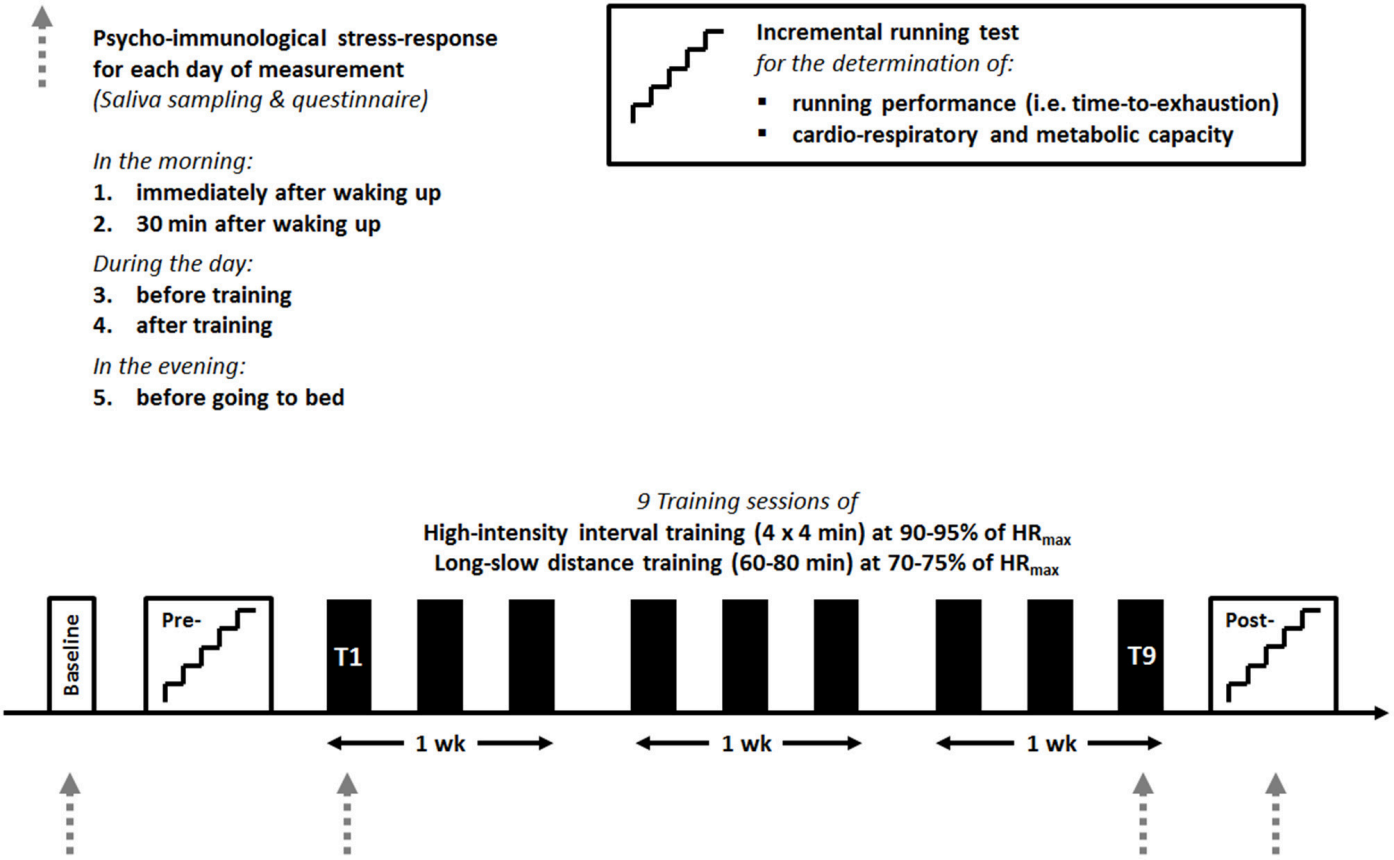
The study design with the time-points for the saliva sampling and questionnaire on the baseline day, the first (T1) and last (T9) day of training and the follow-up for both groups.

In order to investigate the psycho-immunological stress-response to nine sessions of either HIIT or LSD, saliva samples along with questionnaires were taken on a baseline day before the start of the study, the first (T1) and last (T9) day of training. The follow-up measurement was performed on the day of the post-test 4 days after the last day of training (Figure 1).

### Data collection

#### Cardio-respiratory and metabolic response

The incremental running test was performed on a treadmill (H/P Cosmos, Mercury, Nussdorf-Traunstein, Germany) and initiated with a running velocity of 2.4 m/s. Subsequently, the running velocity was increased by 0.4 m/s in 5-min intervals until voluntary exhaustion. The incremental test was performed at 1% inclination to simulate the missing air resistance and drag forces of outdoor running (Gore, 2000). Maximal effort was considered when the runners met three of the following four criteria: (1) V^·^O_2_ showed a leveling-off defined as an increase of V^·^O_2_ of <2.1 mL/kg/min (Taylor et al., 1955), (2) respiratory exchange ratio > 1.05, (3) HR ≥ 90% of the age-predicted HR, (4) ratings of perceived exertion ≥18 on Borg's 6–20 scale (Borg, 1970).

During the incremental running test, the participants were equipped with an open-circuit breath-by-breath gas analyzer (MetaMax3B_R2, Cortex Biophysik GmbH, Leipzig, Germany) breathing through a turbine flowmeter which was attached to a proper fitting face mask covering the mouth and nose (7,450 Series V2 TM Mask, Hans Rudolph Inc., Shawnee, USA). The HR was collected time aligned with the V^·^O_2_ data using a chest belt (H7, Polar Electro Oy, Kempele, Finland). Before each test the oxygen (O_2_) and carbon dioxide (CO_2_) sensors of the gas analyzer were 2-point calibrated to ambient air (20.93% O_2_ and 0.03% CO_2_) and calibration gas containing 15% O_2_ and 5% CO_2_ (UN 1950 Aerosols, Cortex Biophysik GmbH, Leipzig, Germany) to anticipate the expiratory gas compound. The turbine's flow volume was calibrated using a 3-L syringe (M9474-C, Medikro Oy, Kuopio, Finland). The levels of blood lactate concentration were determined in the capillary blood sampled from the left ear lobe (LactatePro2, LT-1730, Arkray, Kyoto, Japan) and used for the subsequent linear extrapolation of the running velocities at 2 and 4 mmol/L blood lactate concentration.

#### Psycho-immunological stress-response

In order to assess the acute exercise induced stress-response as well as the circadian variation of the markers of interest, on each day of measurement (baseline, T1, T9, and follow-up) five saliva samples were taken: (1) immediately after waking up, (2) 30 min after waking up, (3) immediately before training, (4) immediately after training, and (5) before going to bed as described previously (Rohleder et al., 2007; Born et al., 2016).

To collect the saliva samples all athletes received the following instructions: (1) not to eat and drink (other than plain water) or brushing teeth for 30 min beforehand to avoid blood contamination of the saliva sample, (2) rinse the mouth with water and swallow any remaining fluid, (3) start a stopwatch and passively collect saliva in the mouth while resting in a seated position with the head tilted slightly forward for exactly 2 min, (4) spit the accumulated saliva through a sterile polypropylene straw into a polypropylene tube (Sali-Cap Tubes, IBL International, Hamburg, Germany), (5) store the saliva tube in the freezer at −18°C and bring the sample to the lab the next morning for the analysis of cortisol and sIgA, as recommended previously (Granger et al., 2007). For the first sample of the day, the participants were instructed to collect the saliva immediately after waking up when the alarm went off while sitting at the edge of the bed. For the next 30 min the participants were allowed to walk around, take a shower and prepare the breakfast. They however were not allowed to eat, drink or continue sleeping. The exact time of the day for each saliva sample was reported in a specific protocol.

A scaling on the transparent polypropylene tube showed the volume of saliva collected. In case the collected saliva was <1 mL within the first 2 min the athletes continued collecting saliva for another 2 min. The additional collection time was reported in the protocol for the subsequent determination of saliva flow rate. Especially high-intensity exercise reduces the flow rate of saliva by enhancing sympathetic and/or attenuating the parasympathetic activation (Papacosta and Nassis, 2011). Therefore, the saliva flow rate was employed to calculate the secretion rate of sIgA from the absolute concentrations for any further statistical analysis (Allgrove et al., 2008; Papacosta and Nassis, 2011).

Along with the saliva sample, the athletes rated their current mood on a questionnaire adapted by Wilhelm and Schoebi (2007). The items included levels of stress, anxiety, annoyance, happiness, exhaustion, and energy ranked on a Likert scale from (1) not at all to (5) very much. After decoding, the mood was assessed by the sum of all items while a high score indicated vitality and well-being and a low score a suppressed mood state as described previously (Born et al., 2016). The athletes were asked to report any unusual and stressful events including signs of URTI immediately prior to the saliva sampling since a sympathetic stress-response would dramatically affect the concentration of cortisol, saliva flow rate and mood. In only one occasion a participant reported to be upset from such an event (i.e., a private matter that cased him stress but was not related to the study) and the sample from this particular point of measurement was excluded from the analysis.

All saliva samples were centrifuged at 2,000 g for 10 min to separate the firm mucus at the bottom of the tube. The supernatant aqueous fraction was used to analyze the concentration of cortisol and sIgA using commercially available enzyme-immunoassay kits (DRG Instruments, Marburg, Germany). The standard ranges for the determination of cortisol and sIgA were 2–80 ng/mL and 6.9–400 μg/mL with a sensitivity of 0.5 ng/mL and 0.5 μg/mL at the 95% confidence limit, respectively. The intra-assay coefficient of variation (CV) for cortisol and sIgA were 5.2 and 3.6% with an inter-assay CV of 5.7 and 5.5%, respectively. In order to cope with the inter-individual variation in the concentration of cortisol and sIgA secretion rate, the values from the baseline day were used to normalize the data.

### Statistical analysis

The data are presented as mean values ± standard deviations (SD), normal distribution was confirmed with Shapiro-Wilk's test and an alpha-level <0.05 considered as statistically significant. A 2-way analysis of variance (ANOVA) with repeated measure using Fisher's *post-hoc* test was performed to detect significant differences between the training intensity (HIIT vs. LSD) and time-points of measurement. Additionally, effect size (partial eta^2^) and statistical power were calculated for each variable. As suggested previously (Pruessner et al., 2003), the area under the curve with respect to ground level (AUC_G_) was determined for the psycho-immunological stress-response on each day of measurement. Pearson's product moment correlation coefficient was used to identify potential variables that were related to the change in V^·^O_2peak_ and TTE. All data were recorded and prepared using Excel 2010 (Microsoft Corp., Redmond, USA) and analyzed subsequently with Statistical 10.0 (StatSoft Inc., Tulsa, USA).

## Results

### Physiological assessment

The performance data as well as cardio-respiratory and metabolic response to nine sessions of HIIT and LSD are presented in Table 1. While HIIT and LSD increased the TTE from Pre- to Post- (*P* < 0.01), the interaction effect revealed that the HIIT resulted in a longer TTE at Post- compared to the LSD (*P* = 0.02). The V^·^O_2peak_ increased with the HIIT only (interaction effect, *P* = 0.01) and the levels of blood lactate concentration lessened with the LSD only (interaction effect, *P* = 0.01). The running velocities at 2 and 4 mmol/L blood lactate concentration increased with both, HIIT and LSD (*P* < 0.01 and *P* < 0.01, respectively).

**Table 1:** The performance data as well as cardio-respiratory and metabolic response to nine sessions of HIIT compared to LSD (mean ± SD).

		**HIIT**	**LSD**		***F*-value**	***P*-value**	**Partial eta^2^**	**Test power**
Time-to-exhaustion (s)	Pre-	1887 ± 290	1764 ± 221	a)	*F*_(1, 26)_ = 4	n.s.		
	Post-	2129 ± 298^*^+	1861 ± 220+	b)	*F*_(1, 26)_ = 33	*P* < 0.01	0.56	0.99
				c)	*F*_(1, 26)_ = 6	*P* = 0.02	0.19	0.65
Peak oxygen uptake (mL/kg/min)	Pre-	49 ± 4.5	52.4 ± 4.8	a)	*F*_(1, 26)_ = 1	n.s.		
	Post-	51.5 ± 3.9+	51.8 ± 5.3	b)	*F*_(1, 26)_ = 0	n.s.		
				c)	*F*_(1, 26)_ = 8	*P* = 0.01	0.24	0.79
Maximum heart rate (beats/min)	Pre-	196 ± 8	194 ± 8	a)	*F*_(1, 26)_ = 0	n.s.		
	Post-	194 ± 8	195 ± 8	b)	*F*_(1, 26)_ = 3	n.s.		
				c)	*F*_(1, 26)_ = 4	n.s.		
Maximum blood lactate concentration (mmol/L)	Pre-	8 ± 2.2	9.2 ± 2.1	a)	*F*_(1, 26)_ = 0	n.s.		
	Post-	8.7 ± 2.2	8.2 ± 1.7+	b)	*F*_(1, 26)_ = 0	n.s.		
				c)	*F*_(1, 26)_ = 9	*P* = 0.01	0.25	0.81
Velocity (m/s) at 2 mmol/L blood lactate concentration	Pre-	2.6 ± 0.5	2.4 ± 0.4	a)	*F*_(1, 26)_ = 3	n.s.		
	Post-	2.8 ± 0.4+	2.6 ± 0.3+	b)	*F*_(1, 26)_ = 13	*P* < 0.01	0.33	0.93
				c)	*F*_(1, 26)_ = 0	n.s.		
Velocity (m/s) at 4 mmol/L blood lactate concentration	Pre-	3.3 ± 0.4^*^	3 ± 0.3	a)	*F*_(1, 26)_ = 8	*P* = 0.01	0.25	0.80
	Post-	3.5 ± 0.3^*^+	3.1 ± 0.3+	b)	*F*_(1, 26)_ = 20	*P* < 0.01	0.44	0.99
				c)	*F*_(1, 26)_ = 2	n.s.		

*Significant differences were identified with a 2-way ANOVA: training intensity (HIIT vs. LSD) x time (Pre- vs. Post-). HIIT, High-intensity training; LSD, Long-slow distance training*.

*a) Main effect: training intensity (HIIT vs. LSD)*.

*b) Main effect: time (Pre- vs. Post-)*.

*c) Interaction effect: training intensity x time*.

* *Significant difference compared to LSD*.

+ *significant difference compared to Pre-. n.s., not significant*.

### Psycho-immunological stress-response

Table 2 illustrates the detailed analysis of the AUC_G_ for the psycho-immunological stress-response during the first and last day of training and during the follow-up. A main effect for the time was evident as the AUC_G_ for the levels of cortisol increased during the follow-up in both groups (*P* < 0.01). The *post-hoc* analysis revealed that, the levels of cortisol were increased from before to after exercise (*P* < 0.05) and increased compared to the corresponding values at T1 (*P* < 0.05) with both groups on the day of follow-up. The cortisol values normalized by the end of the day however, showing lower values before going to bed compared to immediately after waking up on all days of measurement (i.e., T1, T9, and follow-up) with the HIIT (*P* < 0.01) and LSD (*P* < 0.01).

**Table 2:** The AUC_G_ for the psycho-immunological stress-response to nine sessions of HIIT compared to LSD on the first (T1) and last (T9) day of training as well as the follow-up (mean ± SD).

		**HIIT**	**LSD**		***F*-value**	***P*-value**	**Partial eta^2^**	**Test power**
Saliva flow rate (mL/min)	T1	1476 ± 198	1346 ± 445	a)	*F*_(1, 26)_ = 1	n.s.		
	T9	1461 ± 316	1332 ± 393	b)	*F*_(1, 26)_ = 7	*P* < 0.01	0.21	0.91
	Follow-up	1225 ± 233+#	1157 ± 426	c)	*F*_(1, 26)_ = 0	n.s.		
Levels of cortisol (ng/mL)	T1	1809 ± 384	1554 ± 426	a)	*F*_(1, 26)_ = 1	n.s.		
	T9	1597 ± 565	1394 ± 257	b)	*F*_(1, 26)_ = 11	*P* < 0.01	0.3	0.99
	Follow-up	1941 ± 354#	2104 ± 855+#	c)	*F*_(1, 26)_ = 2	n.s.		
Salivary immunoglobin A secretion rate (μg/min)	T1	1917 ± 1178^*^	1010 ± 506	a)	*F*_(1, 26)_ = 8	*P* = 0.01	0.23	0.77
	T9	2123 ± 1394^*^	1142 ± 628	b)	*F*_(1, 26)_ = 1	n.s.		
	Follow-up	1968 ± 1164^*^	962 ± 427	c)	*F*_(1, 26)_ = 0	n.s.		
Mood (a.u.)	T1	1363 ± 204	1413 ± 150	a)	*F*_(1, 26)_ = 0	n.s.		
	T9	1391 ± 152	1444 ± 180	b)	*F*_(1, 26)_ = 2	n.s.		
	Follow-up	1465 ± 164	1420 ± 92	c)	*F*_(1, 26)_ = 3	n.s.		

*Significant differences were identified with a 2-way ANOVA with repeated measure: training intensity (HIIT vs. LSD) x time (T1 vs. T9 vs. Follow-up)*.

*a) Main effect: training intensity (HIIT vs. LSD)*.

*b) Main effect: time (T1 vs. T9 vs. Follow-up)*.

*c) Interaction effect: training intensity x time*.

* *Significant difference compared to LSD*.

+ *Significant difference compared to T1*.

# *Significant difference compared to T9*.

*n.s., Not significant*.

**Figure 2:**
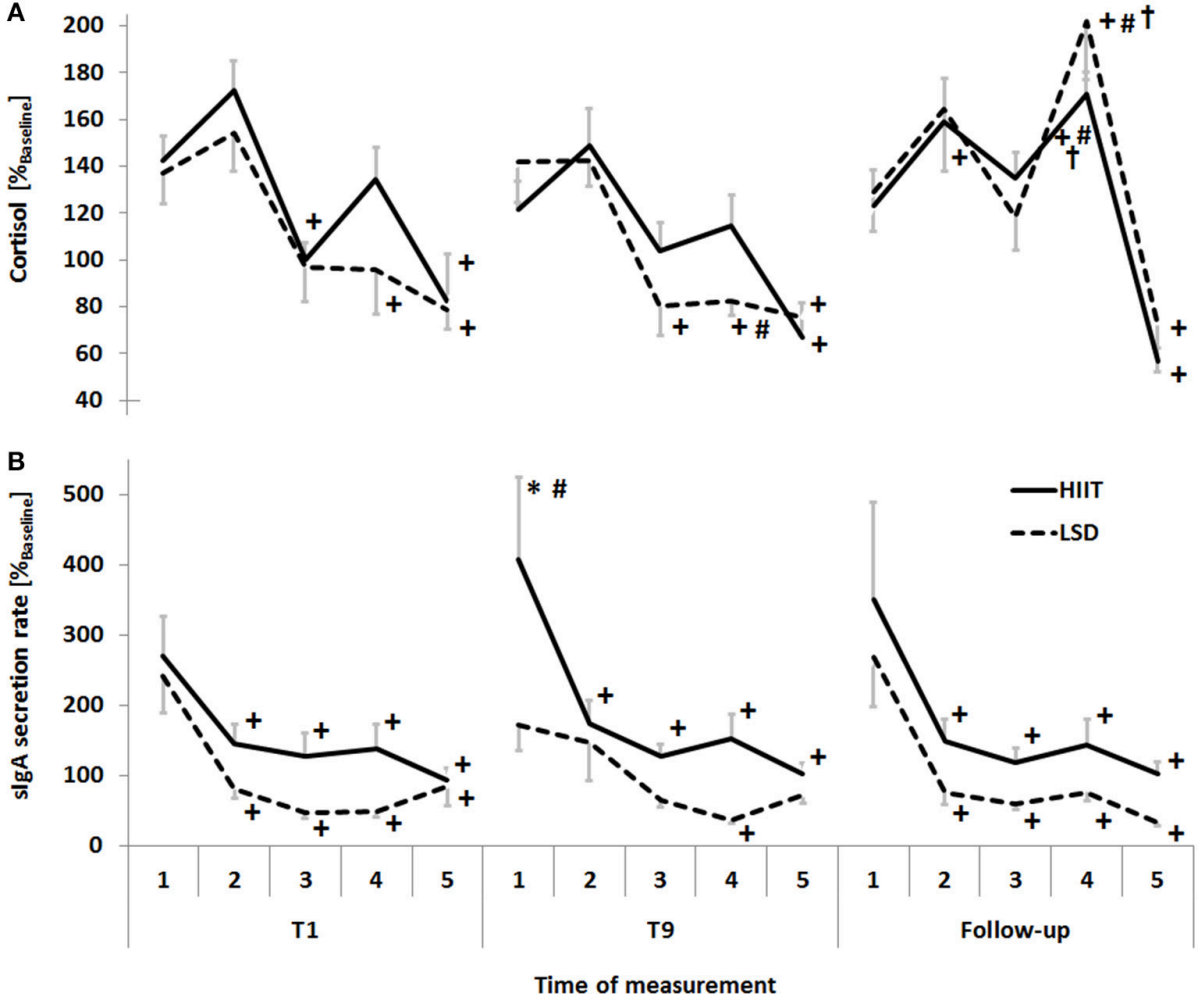
The circadian variation of the **(A)** levels of cortisol and **(B)** salivary immunoglobin A (sIgA) secretion rate in response to the high-intensity interval training (HIIT) and the long-slow distance training (LSD) normalized to the concentrations obtained from the baseline day before the start of the training period. Samples were taken on the first (T1) and ninth (T9) day of training as well as the follow-up (1) immediately after waking up, (2) 30 min after waking up, (3) before training, (4) after training and (5) before going to bed. For the sake of clarity, the standard error is illustrated for the corresponding mean values. Significant differences are indicated as follows: ^*^ between groups, + in comparison to immediately after waking up of the same day, # in comparison to the corresponding value on day T1, † in comparison to before exercise.

A main effect for the training intensity was evident as the AUC_G_ for sIgA secretion rate was higher with the HIIT on T1, T9 and follow-up (*P* = 0.01). The *post-hoc* analysis showed the highest sIgA secretion rate in the morning immediately after waking up with decreasing values throughout the day in both, the HIIT (*P* < 0.05) and LSD (*P* < 0.05). The wake-up response by the end of the training period at T9 was higher with the HIIT compared to the corresponding value at T1 (*P* = 0.01) as well as compared to the LSD (*P* < 0.01; Figure 2). Mood remained unaffected with respect to training intensity and time.

### Correlation analysis

Person correlation analysis detected that, the increased TTE from pre- to post- correlated with the AUC_G_ of sIgA secretion rate on the day of follow-up (*r* = 0.45, *P* = 0.02). As well, the increase in V^·^O_2peak_ was related to the sIgA secretion rate on day T1 (*r* = 0.39, *P* = 0.04).

## Discussion

The main findings of the present study were that, the HIIT results in a longer TTE and increased V^·^O_2peak_ compared to the LSD. The ergogenic effects of HIIT were accompanied with an increased sIgA secretion rate evident as a larger AUC_G_ on the first and last day of training as well as follow-up. The levels of cortisol were unaffected by the training intensity (HIIT vs. LSD) but increased over time on the day of the follow-up with both, HIIT and LSD. Mood remained unaffected with both groups during the entire training period.

The results of the present study are in line with previous findings showing the benefits of HIIT to improve important variables related to the endurance performance, i.e., TTE, and V^·^O_2peak_ (Helgerud et al., 2007; Buchheit and Laursen, 2013a,b; Ronnestad et al., 2014, 2016; Stoggl and Sperlich, 2014, 2015; Sylta et al., 2016). The research focus however was, to investigate the circadian variation of biomarkers in saliva and mood during such a period of HIIT. The question was whether the exposure to nine sessions of HIIT compared to LSD within a 3-week time frame would compromise the mucosal immune function besides the promising effects on TTE and V^·^O_2peak_.

In contrast to the initial hypothesis and the general assumption that chronic exposure to high training loads compromises the mucosal immune function evident as a decreased sIgA secretion rate (Tiollier et al., 2005; Trochimiak and Hubner-Wozniak, 2012) the HIIT in the present investigation actually increased the sIgA secretion rate throughout the entire training period. An earlier review concluded that extreme efforts, such as HIIT, would increase the infection risk of the upper-respiratory tract while moderate intensity exercise, such as LSD, would improve the mucosal immune function (Trochimiak and Hubner-Wozniak, 2012). A “J”-shaped relationship was generally accepted between the immune function and the training load, while both, too low as well as too high training loads, would impair the immune function (Trochimiak and Hubner-Wozniak, 2012). The latter assumption however presumes that the mucosal immune system needs to be stressed in some way to adapt and improve its capacity to neutralize and defend viral pathogens in a similar way as muscles becomes stronger when exposed to regular training stress and adequate overload.

The question arises, how much training and overload is necessary to improve the mucosal immune function. In the present study, the stimulus of nine sessions of HIIT followed by at least 1 day of recovery was adequate to increase the number of antimicrobial proteins in the saliva as indicated by an elevated sIgA secretion rate. In contrast, the LSD did not provide a sufficient stimulus to adapt and augment the mucosal immune function evident as a sIgA secretion rate that was unaltered over the time course of the training period.

From a mechanistic perspective, the sIgA is one of the most abundant antimicrobial proteins in the saliva (Papacosta and Nassis, 2011) and synthesized locally in the submucosa (Allgrove et al., 2008). The activation of the sympathetic nervous system and hypothalamic-pituitary-adrenal-axis promotes the transepithelial transport of sIgA to the mucosal surface (Goodrich and McGee, 1998). Especially the sIgA secretion rate increased acutely immediately after a bout of high-intensity exercise (Allgrove et al., 2008). The concentration of salivary cortisol however did not respond until 1 h after exercise (Allgrove et al., 2008) explaining why the cortisol response remained unaffected in the present study taking the saliva samples immediately after the HIIT and LSD. While the sIgA secretion rate responded immediately to the increased stress of HIIT, sIgA could therefore be a marker of stress being more sensitive than salivary cortisol.

The question remains, whether the increased sIgA secretion rate when waking up on T9 with the HIIT shows the stress response from the previous training session, which was still evident during recovery, or if the increased sIgA secretion rate indicates a chronically enhanced mucosal immune function. In both cases, with the repeated exposure to high training loads (nine sessions of HIIT) the enhanced secretion of sIgA must be matched by an increased synthesis in the submucosal plasma cells (Goodrich and McGee, 1998). Otherwise, over the time course of nine HIIT sessions the store of IgA in the submucosa available for transport across the epithelium would become depleted (Proctor et al., 2003). The lack of any peak elevation of sIgA with the LSD indicates that the more intense training stimulus with the HIIT must has induced functional adaptation that elevated the sIgA synthesis and augmented the mucosal immune function in addition to the ergogenic effects of a longer TTE and increased V^·^O_2peak_.

Recently, particular interest has been drawn to the correlation of reduced sIgA secretion rate and increased risk of URTI. During polarized endurance training including both, continuous and interval training, the runners who showed greater basal sIgA concentrations suffered less from URTI. Interestingly, the sIgA concentration before the start of the study predicted the number of sick-days (*r* = −0.76, *P* < 0.01) during the following 12-week training period (Ihalainen et al., 2016). The aforementioned findings indicate that the mucosal immune function might be predisposed by either genetic factors or affected by the individual's training history. The findings of the present study support the latter by showing an increased sIgA secretion rate in response to the more severe training stress of the HIIT compared to the moderate intensity exercise with the LSD.

Therefore, the generally accepted “J”-shaped relationship between training load and the risk of URTI needs to be questioned. In a recent review Walsh and Oliver (2016) discussed this matter based on the current literature and concluded “*that international athletes performing high-volume training suffer fewer, not greater, URTI episodes than lower-level performers*”. The authors also concluded that the immune function is actually improved with the regular but intermittent exposure to various forms of stress (Walsh and Oliver, 2016). Linked with the results of the present study it might be time to consider the mucosal immune function as a highly adaptable system that, at least in a 3-week time period, responds well to the stress of HIIT.

In the present investigation, salivary cortisol remained unaffected immediately after exercise on the first and last day of training with both training groups. During the day of the follow-up however the incremental exercise test to exhaustion increased the levels of cortisol immediately after exercise with both, the HIIT and LSD. Investigations evidenced that the participation in official competitions induced greater levels of cortisol compared to training matches or race simulations indicating that the psychological stress itself rather than the actual physical load affects the response in cortisol (Rohleder et al., 2007; Moreira et al., 2012a,b, 2013). When comparing two volleyball matches that were played against the same opponent, the more important final championship match induced a greater cortisol response compared to the regular season match (Moreira et al., 2013). Similarly, in elite basketball players the cortisol values were still elevated largely above baseline during the competition phase even with a physical load that was almost half as much as during the preceding training phase (He et al., 2010). In the present study, the incremental exercise test that was performed on the day of the follow-up created a semi-competitive situation since each runner attempted to compel themselves mentally and physically as hard as possible in order to profit from the past training period and outrun their training colleagues. The arousal, anxiety, and pressure to perform well during this type of performance test seemed to induce a substantial cortisol response which was not evident at any time point during the training phase neither with the HIIT nor LSD.

Recent studies showed that the compromised mucosal immune function, i.e., reduced sIgA secretion rate, induced by the stress of competition was accompanied with high levels of cortisol (He et al., 2010; Moreira et al., 2013). Therefore, an antagonistic activity of increased cortisol values that inhibit the sIgA response has been discussed (He et al., 2010). In the present study, especially the HIIT required the runners to perform each training session close to their physical limit at 90–95% of HR_max_. The mood data however show, that our athletes did not feel pressured or psychologically stressed due to the high training loads of HIIT shown by a mood state that was not different to the LSD. Also the levels of cortisol on the first and last day of training were fairly low with the HIIT and not different to LSD. Assuming an antagonistic activity of cortisol and sIgA (He et al., 2010; Moreira et al., 2013), the low levels of cortisol on the first and last day of training could explain why our athletes did not show any reduced sIgA secretion rate with the HIIT but adapted to the training stress and improved their mucosal immune function.

## Conclusion

In contrast to the hypothesis, we could not investigate any signs of a compromised mucosal immune function with the HIIT compared to LSD. The ergogenic effects of HIIT, i.e., increased V^·^O_2peak_, were even accompanied with an increased sIgA secretion rate indicating that the mucosal immune system adapted over the time course of the training period by increasing the number of antimicrobial proteins and improving the capacity to neutralize and defend viral pathogens. The training stimulus of the LSD on the other hand was insufficient to improve the mucosal immune function or V^·^O_2peak_. Based on our data we cannot generally accept the assumption that high training loads necessarily compromises the mucosal immune function. Connecting the data of the present study with previous findings (Born et al., 2016; Walsh and Oliver, 2016), it might be time to consider the mucosal immune function as a highly adaptable system that responds well to the stress and load of training, in particular nine sessions of HIIT within 3 weeks.

The HIIT had no effect on the levels of cortisol and mood. Therefore, the psychological stress, i.e., the arousal, anxiety, mental stress, and pressure to perform well during competition (Rohleder et al., 2007; Moreira et al., 2012a,b, 2013), rather than the actual physical load of exercise might be responsible for an impaired mucosal immune function. Future studies should apply the circadian variation of sIgA secretion rate, cortisol and mood to further distinguish between the psychological and physical stressors and how both could impact the mucosal immune function during periods with an intensified training load and competition.

## Author contributions

Conception of the experimental design, data collection, analysis, interpretation, preparing and critically revising the manuscript: DB, CZ, and BS. All authors read and approved the final version of the manuscript.

### Conflict of interest statement

The authors declare that the research was conducted in the absence of any commercial or financial relationships that could be construed as a potential conflict of interest.

## References

[B1] Allgrove J. E. Gomes E. Hough J. Gleeson M. (2008). Effects of exercise intensity on salivary antimicrobial proteins and markers of stress in active men. J. Sports Sci. 26, 653–661. 10.1080/02640410701716790 18344136

[B2] Borg G. (1970). Perceived exertion as an indicator of somatic stress. Scand. J. Rehabil. Med. 2, 92–98. 5523831

[B3] Born D. P. Faiss R. Willis S. J. Strahler J. Millet G. P. Holmberg H. C.. (2016). Circadian variation of salivary immunoglobin A, alpha-amylase activity and mood in response to repeated double-poling sprints in hypoxia. Eur. J. Appl. Physiol. 116, 1–10. 10.1007/s00421-015-3236-3 26269448

[B4] Buchheit M. Laursen P. B. (2013a). High-intensity interval training, solutions to the programming puzzle. Part II: anaerobic energy, neuromuscular load and practical applications. Sports Med. 43, 927–954. 10.1007/s40279-013-0066-5 23832851

[B5] Buchheit M. Laursen P. B. (2013b). High-intensity interval training, solutions to the programming puzzle: Part I: cardiopulmonary emphasis. Sports Med. 43, 313–338. 10.1007/s40279-013-0029-x 23539308

[B6] Cadore E. Lhullier F. Brentano M. Silva E. Ambrosini M. Spinelli R.. (2008). Correlations between serum and salivary hormonal concentrations in response to resistance exercise. J. Sports Sci. 26, 1067–1072. 10.1080/02640410801919526 18608830

[B7] Faiss R. Willis S. Born D. Sperlich B. Vesin J. Holmberg H.. (2015). Double-poling repeated sprint training in hypoxia by competitive cross-country skiers. Med. Sci. Sports Exerc. 47, 809–817. 10.1249/MSS.0000000000000464 25083727

[B8] Gatti R. De Palo E. F. (2011). An update: salivary hormones and physical exercise. Scand. J. Med. Sci. Sports 21, 157–169. 10.1111/j.1600-0838.2010.01252.x 21129038

[B9] Gill S. K. Teixeira A. M. Rosado F. Hankey J. Wright A. Marczak S.. (2014). The impact of a 24-h ultra-marathon on salivary antimicrobial protein responses. Int. J. Sports Med. 35, 966–971. 10.1055/s-0033-1358479 24886918

[B10] Gleeson M. (2007). Immune function in sport and exercise. J. Appl. Physiol. 103, 693–699. 10.1152/japplphysiol.00008.2007 17303714

[B11] Goodrich M. E. McGee D. W. (1998). Regulation of mucosal B cell immunoglobulin secretion by intestinal epithelial cell-derived cytokines. Cytokine 10, 948–955. 10.1006/cyto.1998.0385 10049518

[B12] Gore C. J. (2000). Physiological Tests for Elite Athletes. Leeds: Human Kinetics.

[B13] Granger D. A. Kivlighan K. T. Fortunato C. Harmon A. G. Hibel L. C. Schwartz E. B.. (2007). Integration of salivary biomarkers into developmental and behaviorally-oriented research: problems and solutions for collecting specimens. Physiol. Behav. 92, 583–590. 10.1016/j.physbeh.2007.05.004 17572453

[B14] He C. S. Tsai M. L. Ko M. H. Chang C. K. Fang S. H. (2010). Relationships among salivary immunoglobulin A, lactoferrin and cortisol in basketball players during a basketball season. Eur. J. Appl. Physiol. 110, 989–995. 10.1007/s00421-010-1574-8 20668874

[B15] Helgerud J. Hoydal K. Wang E. Karlsen T. Berg P. Bjerkaas M.. (2007). Aerobic high-intensity intervals improve VO2max more than moderate training. Med. Sci. Sports Exerc. 39, 665–671. 10.1249/mss.0b013e3180304570 17414804

[B16] Hucklebridge F. Clow A. Evans P. (1998). The relationship between salivary secretory immunoglobulin A and cortisol: neuroendocrine response to awakening and the diurnal cycle. Int. J. Psychophysiol. 31, 69–76. 10.1016/S0167-8760(98)00042-7 9934622

[B17] Hydren J. R. Cohen B. S. (2015). Current scientific evidence for a polarized cardiovascular endurance training model. J. Strength Cond. Res. 29, 3523–3530. 10.1519/JSC.0000000000001197 26595137

[B18] Ihalainen J. K. Schumann M. Hakkinen K. Mero A. A. (2016). Mucosal immunity and upper respiratory tract symptoms in recreational endurance runners. Appl. Physiol. Nutr. Metab. 41, 96–102. 10.1139/apnm-2015-0242 26701121

[B19] Moreira A. Crewther B. Freitas C. G. Arruda A. F. Costa E. C. Aoki M. S. (2012a). Session RPE and salivary immune-endocrine responses to simulated and official basketball matches in elite young male athletes. J. Sports Med. Phys. Fitness 52, 682–687. 23187333

[B20] Moreira A. Franchini E. de Freitas C. G. Schultz de Arruda A. F. de Moura N. R. Costa E. C.. (2012b). Salivary cortisol and immunoglobulin A responses to simulated and official Jiu-Jitsu matches. J. Strength Cond. Res. 26, 2185–2191. 10.1519/JSC.0b013e31823b8702 22027851

[B21] Moreira A. Freitas C. G. Nakamura F. Y. Drago G. Drago M. Aoki M. S. (2013). Effect of match importance on salivary cortisol and immunoglobulin A responses in elite young volleyball players. J. Strength Cond. Res. 27, 202–207. 10.1519/JSC.0b013e31825183d9 22395269

[B22] Ni Cheilleachair N. J. Harrison A. J. Warrington G. D. (2017). HIIT enhances endurance performance and aerobic characteristics more than high-volume training in trained rowers. J. Sports Sci. 35, 1052–1058. 10.1080/02640414.2016.1209539 27438378

[B23] Papacosta E. Gleeson M. Nassis G. P. (2013). Salivary hormones, IgA, and performance during intense training and tapering in judo athletes. J. Strength Cond. Res. 27, 2569–2580. 10.1519/JSC.0b013e31827fd85c 23249825

[B24] Papacosta E. Nassis G. P. (2011). Saliva as a tool for monitoring steroid, peptide and immune markers in sport and exercise science. J. Sci. Med. Sport 14, 424–434. 10.1016/j.jsams.2011.03.004 21474377

[B25] Proctor G. B. Garrett J. R. Carpenter G. H. Ebersole L. E. (2003). Salivary secretion of immunoglobulin A by submandibular glands in response to autonomimetic infusions in anaesthetised rats. J. Neuroimmunol. 136, 17–24. 10.1016/S0165-5728(02)00466-6 12620639

[B26] Pruessner J. C. Kirschbaum C. Meinlschmid G. Hellhammer D. H. (2003). Two formulas for computation of the area under the curve represent measures of total hormone concentration versus time-dependent change. Psychoneuroendocrinology 28, 916–931. 10.1016/S0306-4530(02)00108-7 12892658

[B27] Rohleder N. Beulen S. E. Chen E. Wolf J. M. Kirschbaum C. (2007). Stress on the dance floor: the cortisol stress response to social-evaluative threat in competitive ballroom dancers. Pers. Soc. Psychol. Bull. 33, 69–84. 10.1177/0146167206293986 17178931

[B28] Ronnestad B. R. Hansen J. Ellefsen S. (2014). Block periodization of high-intensity aerobic intervals provides superior training effects in trained cyclists. Scand. J. Med. Sci. Sports 24, 34–42. 10.1111/j.1600-0838.2012.01485.x 22646668

[B29] Ronnestad B. R. Hansen J. Thyli V. Bakken T. A. Sandbakk O. (2016). 5-week block periodization increases aerobic power in elite cross-country skiers. Scand. J. Med. Sci. Sports 26, 140–146. 10.1111/sms.12418 25648345

[B30] Stoggl T. L. Sperlich B. (2015). The training intensity distribution among well-trained and elite endurance athletes. Front. Physiol. 6:295. 10.3389/fphys.2015.00295 26578968 PMC4621419

[B31] Stoggl T. Sperlich B. (2014). Polarized training has greater impact on key endurance variables than threshold, high intensity, or high volume training. Front. Physiol. 5:33. 10.3389/fphys.2014.00033 24550842 PMC3912323

[B32] Sylta O. Tonnessen E. Hammarstrom D. Danielsen J. Skovereng K. Ravn T.. (2016). The effect of different high-intensity periodization models on endurance adaptations. Med. Sci. Sports Exerc. 48, 2165–2174. 10.1249/MSS.0000000000001007 27300278

[B33] Tanner A. V. Nielsen B. V. Allgrove J. (2014). Salivary and plasma cortisol and testosterone responses to interval and tempo runs and a bodyweight-only circuit session in endurance-trained men. J. Sports Sci. 32, 680–689. 10.1080/02640414.2013.850594 24279436

[B34] Taylor H. L. Buskirk E. Henschel A. (1955). Maximal oxygen intake as an objective measure of cardio-respiratory performance. J. Appl. Physiol. 8, 73–80. 13242493 10.1152/jappl.1955.8.1.73

[B35] Tiollier E. Gomez-Merino D. Burnat P. Jouanin J. C. Bourrilhon C. Filaire E.. (2005). Intense training: mucosal immunity and incidence of respiratory infections. Eur. J. Appl. Physiol. 93, 421–428. 10.1007/s00421-004-1231-1 15490219

[B36] Trochimiak T. Hubner-Wozniak E. (2012). Effect of exercise on the level of immunoglobulin a in saliva. Biol. Sport 29, 255–261. 10.5604/20831862.1019662 24868115 PMC4033058

[B37] VanBruggen M. D. Hackney A. C. McMurray R. G. Ondrak K. S. (2011). The relationship between serum and salivary cortisol levels in response to different intensities of exercise. Int. J. Sports Physiol. Perform. 6, 396–407. 10.1123/ijspp.6.3.396 21911864

[B38] Walsh N. P. Oliver S. J. (2016). Exercise, immune function and respiratory infection: an update on the influence of training and environmental stress. Immunol. Cell Biol. 94, 132–139. 10.1038/icb.2015.99 26563736

[B39] Wilhelm P. Schoebi D. (2007). Assessing mood in daily life - Structural validity, sensitivity to change, and reliability of a short-scale to measure three basic dimensions of mood. Eur. J. Psychol. Assess. 23, 258–267. 10.1027/1015-5759.23.4.258

